# H5N1 and 1918 Pandemic Influenza Virus Infection Results in Early and Excessive Infiltration of Macrophages and Neutrophils in the Lungs of Mice

**DOI:** 10.1371/journal.ppat.1000115

**Published:** 2008-08-01

**Authors:** Lucy A. Perrone, Julie K. Plowden, Adolfo García-Sastre, Jacqueline M. Katz, Terrence M. Tumpey

**Affiliations:** 1 Immunology and Pathogenesis Branch, Influenza Division, National Center for Immunization and Respiratory Diseases, Collaborating Centers for Infectious Diseases, Centers for Disease Control and Prevention, Atlanta, Georgia, United States of America; 2 Department of Microbiology, Mount Sinai School of Medicine, New York, New York, United States of America; 3 Department of Medicine, Division of Infectious Diseases, Mount Sinai School of Medicine, New York, New York, United States of America; 4 Emerging Pathogens Institute, Mount Sinai School of Medicine, New York, New York, United States of America; University of North Carolina, United States of America

## Abstract

Fatal human respiratory disease associated with the 1918 pandemic influenza virus and potentially pandemic H5N1 viruses is characterized by severe lung pathology, including pulmonary edema and extensive inflammatory infiltrate. Here, we quantified the cellular immune response to infection in the mouse lung by flow cytometry and demonstrate that mice infected with highly pathogenic (HP) H1N1 and H5N1 influenza viruses exhibit significantly high numbers of macrophages and neutrophils in the lungs compared to mice infected with low pathogenic (LP) viruses. Mice infected with the 1918 pandemic virus and a recent H5N1 human isolate show considerable similarities in overall lung cellularity, lung immune cell sub-population composition and cellular immune temporal dynamics. Interestingly, while these similarities were observed, the HP H5N1 virus consistently elicited significantly higher levels of pro-inflammatory cytokines in whole lungs and primary human macrophages, revealing a potentially critical difference in the pathogenesis of H5N1 infections. These results together show that infection with HP influenza viruses such as H5N1 and the 1918 pandemic virus leads to a rapid cell recruitment of macrophages and neutrophils into the lungs, suggesting that these cells play a role in acute lung inflammation associated with HP influenza virus infection. In addition, primary macrophages and dendritic cells were also susceptible to 1918 and H5N1 influenza virus infection *in vitro* and in infected mouse lung tissue.

## Author Summary

Patients who succumbed to influenza during the 1918 pandemic had severe lung pathology marked by extensive inflammatory infiltrate, indicating a robust immune response in the lung. Similar findings have been reported from H5N1-infected patients, raising the question as to why people expire in the presence of a strong immune response. We addressed this question by characterizing the immune cell populations in the mouse lung following infection with the 1918 pandemic virus and two H5N1 viruses isolated from fatal cases. Our data shows excessive immune cell infiltration in the lungs contributing to severe consolidation and tissue architecture destruction in mice infected with highly pathogenic (HP) influenza viruses, supporting the histopathological observations of lung tissue from 1918 and H5N1 fatalities. We found that certain cells of the innate immune system, specifically macrophages and neutrophils, increase significantly into the mouse lung shortly following HP virus infection. Interestingly, lung macrophages and dendritic cells were shown to be susceptible to 1918 and H5N1 virus infection *in vitro* or *ex vivo*, suggesting a possible mechanism of immunopathogenesis. Identification of the precise inflammatory cells associated with lung inflammation will be important for the development of treatments that could potentially enhance or modulate host innate immune responses.

## Introduction

The influenza pandemic from 1918 to 1919 was the most devastating infectious disease pandemic ever documented in such a short period of time, killing nearly 50 million people worldwide [1]. Unlike the epidemiological profiles of most influenza infections, young adults aged 18–35 yrs old had the highest mortality rate, so much so that the average life expectancy during those years was lowered by 10 years [2]. In 1918, severe destruction of lung tissue observed by pathologists at autopsy was unlike that typically seen in cases of pneumonia [3] and histopathological analysis of lung tissue showed severe tissue consolidation with unique destruction of the lung architecture [3],[4]. Human infections with highly pathogenic avian influenza (HPAI) strains of subtype H5N1 since the first outbreak in 1997 have also been particularly severe for children and young adults [5]–[7]. Assessing pulmonary infiltrates in response to influenza H5N1 virus infection has been difficult due to the lack of autopsy material. The basis for the high morbidity and mortality associated with the 1918 virus and recent H5N1 viruses remains inconclusive based on viral genetic analysis alone and accounts of patient lung pathology provide only qualitative information about the host factors contributing to disease [4],[8],[9]. Great concern about a pandemic caused by a novel avian H5 subtype virus warrants comparative studies to better understand the cellular pathology caused by a pandemic virus and potentially pandemic viruses. Identification and quantification of the inflammatory cell types associated with highly pathogenic respiratory infections represent prospective targets for modulation of host innate immune responses.

Recent studies using animal models to investigate the mechanism(s) of severe influenza virulence have implicated the innate immune system in complicating lung tissue recovery [10]–[13]. Mouse models of highly pathogenic (HP) H5N1 [14]–[18] and 1918 [19],[20] influenza virus infection confirm histological observations of severe lung pathology in human patients, however, the types of immune cells present during the peak of lung pathology have not been fully elucidated. Excessive immune cell infiltration during an acute lung injury may impair tissue restoration directly by interfering with gas exchange, or indirectly through the release of soluble immune mediators. In the present study, we determined key immune cellular components in the murine lung following infection with matched H5N1 and H1N1 virus pairs that represent high and low virulence infections of each influenza subtype as previously determined in the mouse model [18],[21]. The two H5N1 viruses used in this study (A/Thailand/16/2004 and A/Thailand/SP/83/2004) were isolated in 2004 from fatal human cases in Thailand but have a differential pathogenic outcome in mice, specifically a low and high mouse lethal does 50 (LD_50_ = 1.7 and 5.6 log_10_ PFU respectively) [18]. For relevant comparison, we also used a contemporary (non lethal) seasonal H1N1 human isolate from 1991 (A/TX/36/91) and the reconstructed 1918 pandemic virus [21]. A detailed flow cytometry evaluation of lung cells demonstrated that macrophages and neutrophils are the prominent cell types associated with and potentially mediating the severe lung pathology following infection with the highly virulent H5N1 and 1918 viruses. Moreover, inoculation of macrophages and dendritic cells with the HP viruses *in vitro* or *ex vivo* reveals that some innate immune cells can themselves serve as targets of viral infection.

## Results

### Highly pathogenic H1N1 and H5N1 viruses exhibit early and sustained replication in murine lung tissue following intranasal infection

**Figure 1:**
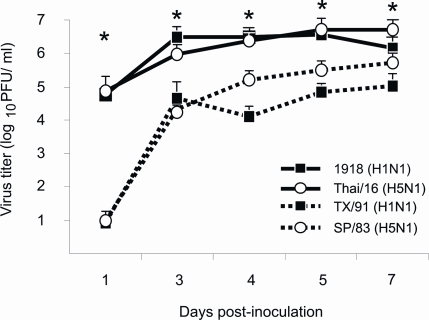
Lung virus titers. Female BALB/c mice were infected intranasally with 10^2^ PFU of influenza viruses and lungs were harvested for virus titration at various times post-inoculation. Lungs were homogenized in 1 ml of PBS and virus titers determined by plaque assay (+ TPCK trypsin 1 µg/ml) on MDCK cells in duplicate (n = 3 mice per time point). * *p*<0.05 between 1918 and Thai/16 infected lungs and TX/91 and SP/83 infected lungs.

Female BALB/c mice were infected intranasally with either highly pathogenic (HP) or low-pathogenic (LP) influenza viruses (Table 1, Methods) based on known LD_50_'s and phenotypes of disease in mouse [21] and ferret [18],[22] models. As shown in Figure 1, the 1918 (H1N1) and A/Thailand/16/2004 (Thai/16, H5N1) viruses replicated to high titers that were at least 5-fold higher than the respective LP viruses, A/Texas/36/91 (TX/91, H1N1) and A/Thailand/SP/83/2004 (SP/83, H5N1) as early as 1 day post-infection (p.i.) and sustained higher levels of replication than the LP viruses throughout the course of infection. 1918 and TX/91 virus infected lungs reached peak titers on day 3 p.i. and remained elevated for the duration of the study. The HP Thai/16 virus reached peak titers later than the 1918 virus at day 5 pi. while the less mouse-virulent SP/83 virus reached it's highest titers on day 7 p.i.. Lung virus titers were significantly higher (*p<0.05) in the 1918 and Thai/16 virus groups compared to TX/91 and SP/83 infected mice at all time points measured.

**Table 1:** Viruses used in this study.

Virus†	Subtype	Source	MLD*50‡	Ferret pathotype‡
A/Texas/36/91	H1N1	Non fatal case	NL	Low virulence
A/1918	H1N1	rgˆ	3.2	High virulence
A/Thailand/SP/83/2004	H5N1	Fatal case, 58 yrs old	5.5	Low virulence
A/Thailand/16/2004	H5N1	Fatal case, 7 yrs old	1.7	High virulence

**†:** Abbreviations used in the text: TX/91, 1918, SP/83, Thai/16

*: Fifty-percent mouse lethal dose (log10) titers.

**‡:** NL = Not mouse lethal [18],[21],[22].

ˆ Generated by reverse genetics [21].

### Increased cellularity in lungs of mice infected with highly pathogenic H1N1 and H5N1 influenza viruses

**Figure 2:**
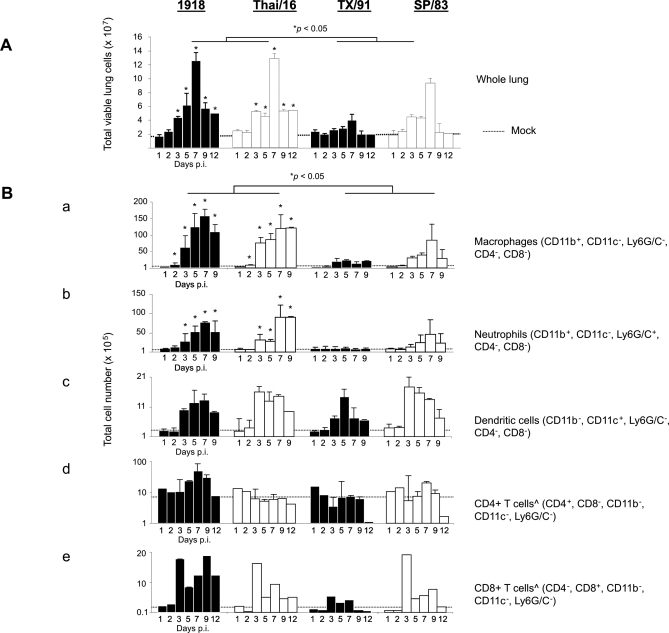
Lung cell characterization following infection with highly pathogenic influenza viruses. (A) Total viable lung cell numbers post-infection. Mice were infected with 10^2^ PFU of influenza viruses, lungs removed and cell suspensions prepared at various times post-inoculation. Viable cells were counted on a hemocytometer by trypan blue exclusion. The data shown represents the total number of viable lung cells from three mice per time point, (per virus group) and standard deviations from the mean cell numbers (× 10^7^ cells) are shown as error bars, * *p*<0.05. (B) Lung immune cell sub-populations. Mean lung immune cell sub-populations (as determined by appropriate gating on labeled cells) are representative of 3 mouse lungs per time point/per virus group and standard deviations are shown in parentheses where available. Numbers of macrophages (CD11b^+^, CD11c^−^, Ly6G/c^−^)(a), neutrophils (CD11b^+^, CD11c^−^, Ly6G/c^+^) (b), and dendritic cells (CD11b^−^, CD11c^+^, Ly6G/c^−^) (c) were determined by appropriate gating within the total lung leukocytes. T cell numbers (d and e) were determined by gating within the lymphocyte gate of the total leukocyte population gate. ˆ On days 1, 2 and 12 p.i (CD 4^+^) and CD8^+^ cells (all time points) are presented as averages of 3 lungs.

Whole lungs collected (without perfusion, therefore including the bronchoalveolar lavage contents (BAL)) from both 1918 and Thai/16 virus-infected mice showed an increase in overall tissue cellularity as early as 3 days p.i. (Figure 2A). By day 3 p.i., lungs infected with the HP influenza viruses (containing between 4.3–5.1×10^7^ cells) had nearly twice as many total lung cells than were measured in the HP infection groups just 24 hours previously at day 2 p.i. (2.1–2.4×10^7^ cells). Significant differences (*p<0.05) were observed between HP and LP infection groups in total lung cell number at every time after day 2 p.i. Total lung cell numbers doubled in HP infection groups between days 5 and 7 p.i. and by day 7 p.i., when viral titers were high for all four viruses, total cell numbers in the lungs of mice infected with either HP H1N1 or H5N1 viruses were 6-fold higher than those in PBS-inoculated mice, and at least 3-fold higher than those found in LP virus-infected lungs (Figures 1 and 2A). On day 7 p.i., there were as many as 1.3×10^8^ cells in HP-infected lungs compared with 4.0–8.0×10^7^ cells in LP-infected and 1.8×10^7^ cells in PBS- inoculated lungs.

To quantify the immune cell sub-populations responding to viral infection, we next determined the total cell numbers of specific inflammatory cell populations in the infected lungs using flow cytometry (Figure 2B). Compared with PBS- inoculated animals, mice infected with each of the viruses exhibited an increase in the numbers of macrophages (CD11b^+^, CD11c^−^, Ly6G/c^−^, CD4^−^, CD8^−^) beginning 3 days p.i. and continuing through day 9 p.i. Strikingly, there were significantly (*p<0.05) more macrophages in HP virus-infected lungs than in LP virus-infected lungs from days 2 through 9 p.i. (Figure 2B, a). As early as 2 days p.i, there was 1–2 million more macrophages in the lungs of HP-infected lungs compared to LP infected lungs. 1918 and Thai/16 virus–infected lungs had twice as many and nearly 4 times as many macrophages compared to LP viruses at days 3 and 5, respectively. Numbers of lung macrophages peaked in 1918 and Thai/16 infected mice (1.5 and 1.2×10^7^ cells, respectively) at day 7 p.i. before waning as demonstrated at day 9 p.i.

An increase of at least twice as many neutrophils (CD11b^+^, CD11c^−^, Ly6G/c^+^, CD4^−^, CD8-) was observed as early as 1 day p.i. in all infection groups, compared with neutrophils numbers in PBS-inoculated mice. On day 2 p.i. there was on average (n = 3 mice) over four hundred thousand more neutrophils in the lungs of 1918 virus inoculated mice than TX/91 inoculated mice. Significant differences in neutrophil populations (*p<0.05) between the HP and the LP virus groups emerged on day 3 p.i. and were sustained at each subsequent time point measured (Figure 2B, b). On day 3 p.i., more than twice as many neutrophils were found in HP compared to LP infected lungs. Between days 3 and 7 p.i., lungs infected with HP viruses displayed a three fold increase in neutrophil numbers. At the peak of neutrophil infiltration on day 7 p.i, there was 4–8 million more neutrophils in the lungs of HP-infected animals compared to LP virus-infected mice. Although numbers of dendritic cells (CD11b^−^, CD11c^+^, Ly6G/c^−^, CD4^−^ , CD8^−^) (Figure 2B, c) and CD4^ +^ T (CD11b^−^, CD11c^+^, Ly6G/c^−^, CD8^−^) and CD8^+^ T (CD11b^−^, CD11c^+^, Ly6G/c^−^, CD4^−^) cells (Figure 2B, d and e) in all groups of infected mice increased slightly compared with numbers in PBS-inoculated animals during the course of infection, no significant differences were found between HP and LP infection groups, suggesting that these cell populations are not major contributors to the histopathological consolidation observed in lungs during HP H5N1 virus infections in mice [16].

### Macrophages and neutrophils account for the highest percentage of the total lung leukocytes following infection with highly pathogenic influenza viruses

We next determined specific immune cell sub-populations given by percent of the total lung leukocyte population in order to reveal differential population dynamics amongst the immune cell populations measured in these studies (Table 2). Strikingly, macrophage populations by day 3 pi, represented 24% and 24.4% (nearly one-quarter) of the total gated lung leukocytes of mice infected with 1918 and Thai/16 (HP) viruses, respectively, which was significantly higher than the frequency of macrophages detected in TX/91 and SP/83 virus-infected mice from days 2 though 9 p.i. (ˆ *p*<0.005). Neutrophil populations were elevated in all infection groups compared to mock levels beginning 1 day p.i (Figure 2, panel B) however percentages of neutrophils in HP virus infected mice were significantly higher (* *p*<0.05) than those in LP virus-infected mice beginning day 2 p.i. and levels remained elevated at each subsequent time point measured (Table 2).

**Table 2:** Mouse lung immune cell population dynamics during influenza virus infection.

Group	Mean % Total Gated Leukocytes (SD‡)							
	Days post-infection							
	1	2	3	5	7	9	12	
1918	5.6 (0.8)	9.9 (1.4)ˆ	24.0 (4.7) * ˆ ±	23.1 (1.2)* ˆ ±	25.6 (1.6)* ˆ ±	21.0 (1.4)* ˆ ±		
TX/91	5.6 (0.2)	6.0 (1.0)	13.7 (5.0)	9.4 (0.9)	8.1 (0.4)	12.6 (1.8)	NA†	Macrophages (CD11b+, CD11c−, Ly6G/C−, CD4−, CD8−)
SP/83	4.6 (0.9)	6.6 (1.6)	14.1 (2.8)	11.1 (1.3)	10.9 (0.3)	9.5 (1.1)		
Thai/16	4.7 (0.7)	10.2 (1.8)	24.4 (6.2)	23.3 (5.3)	22.9 (4.0)	25.2 (0.0)		
PBS	4.9 (0.3)							
1918	7.7 (1.0)	11.9 (0.2)* ˆ	11.4 (3.1)* ˆ	10.1 (0.5)* ˆ	12.8 (1.1)* ˆ ±<</noteref>	10.1 (3.9)* ˆ ±<</noteref>		
TX/91	6.9 (0.4)	7.8 (0.3)	6.1 (0.6)	5.5 (0.7)	5.7 (0.5)	5.6 (0.6)	NA	Neutrophils (CD11b+, CD11c−, Ly6G/C+, CD4−, CD8−)
SP/83	7.7 (0.5)	7.5 (2.5)	6.3 (1.5)	7.8 (4.5)	8.4 (5.9)	8.7 (0.8)		
Thai/16	9.7 (1.1)	8.9 (0.8)	9.3 (3.0)	9.1 (1.3)	15.2 (1.6)	18.8 (0.0)		
PBS	5.3 (0.8)							
1918	3.0 (0.3)	2.5 (0.3)	3.0 (0.4)*	2.3 (0.3)*	2.2 (0.1)*	1.9 (0.2)*		
TX/91	3.5 (1.2)	3.4 (0.5)	4.7 (0.4)	5.7 (1.0)	3.4 (0.6)	3.7 (0.5)	NA	Dendritic cells (CD11b−, CD11c+, Ly6G/C−, CD4−, CD8−)
SP/83	3.7 (0.8)	4.1 (1.0)	6.2 (1.7)	4.4 (0.4)	3.7 (0.6)	3.6 (0.6)		
Thai/16	3.2 (0.1)	4.0 (0.4)	4.6 (1.3)	3.4 (0.6)	2.5 (0.4)	2.0 (0.0)		
PBS	3.8 ( 0.2)							
1918	13.4	12.1	5.7 (2.5)	8.3 (0.8)	4.9 (2.7)	10.6 (2.7)	7.2	
TX/91	11.4	9.2	4.0	6.7 (0.1)	2.5 (0.1)	8.6 (0.6)	2.2	CD4+ T cells (CD4+, CD8−, CD11b−, CD11c−, Ly6G/C−)
SP/83	10.0	14.6	4.9 (3.2)	8.0 (1.6)	3.0 (0.7)	9.6 (1.2)	3.1	
Thai/16	10.2	14.1	3.7 (1.0)	5.2 (0.8)	0.7 (0.3)	4.8	3.9	
PBS	7.2 (2.2)							
1918	3.9	5.4	1.8	4.5	2.6	6.8	4.8	
TX/91	2.1	1.1	2.9	4.3	2.5	1.0	0.8	CD8+ T cells (CD4−, CD8+, CD11b−, CD11c−, Ly6G/C−)
SP/83	2.3	1.4	2.3	2.2	1.8	5.9	1.5	
Thai/16	2.2	2.8	2.7	3.0	1.7	1.3	1.6	
PBS	2.0							

BALB/c mice were infected intra-nasally with 102 PFU influenza viruses (Table 1). Lung immune cell populations were analyzed by flow cytometry from single cell suspensions from three mice per time point, per virus group.

**‡:** Numbers of immune cell sub-populations are represented here as percent of the total leukocytes measured in these assays. Standard deviations are shown in parenthesis where available. PBS group % populations represented are daily averages.

*: p<0.05 between HP (1918, Thai/16) and LP (TX/91,SP/83) infection groups.

ˆ p<0.05 between 1918 and TX/91 infection groups.

**±:** p<0.05 between Thai/16 and SP/83 infection groups.

**†:** Not analyzed.

In contrast, dendritic cell populations as percent of the total leukocyte population declined over the course of infection with HP viruses while percentages increased in LP infections, peaking at days 3 and 5 p.i. (Table 2). Significant differences in percent dendritic cells between HP and LP infection groups were observed day 3 p.i. and at all other subsequent time points (* *p*<0.05). Percentages of CD4^+^ T cells in the lungs decreased in all infection groups but no significant differences were observed in CD4^+^ or CD8^+^ T cell population dynamics between HP and LP infection groups. Together, these results indicate that macrophages and neutrophils are responsible for the majority increase in total lung cell numbers following infection with the 1918 and HP H5N1 influenza viruses.

### The 1918 pandemic virus and a highly pathogenic H5N1 virus elicit significantly high levels of pro-inflammatory cytokines in the lung

**Figure 3:**
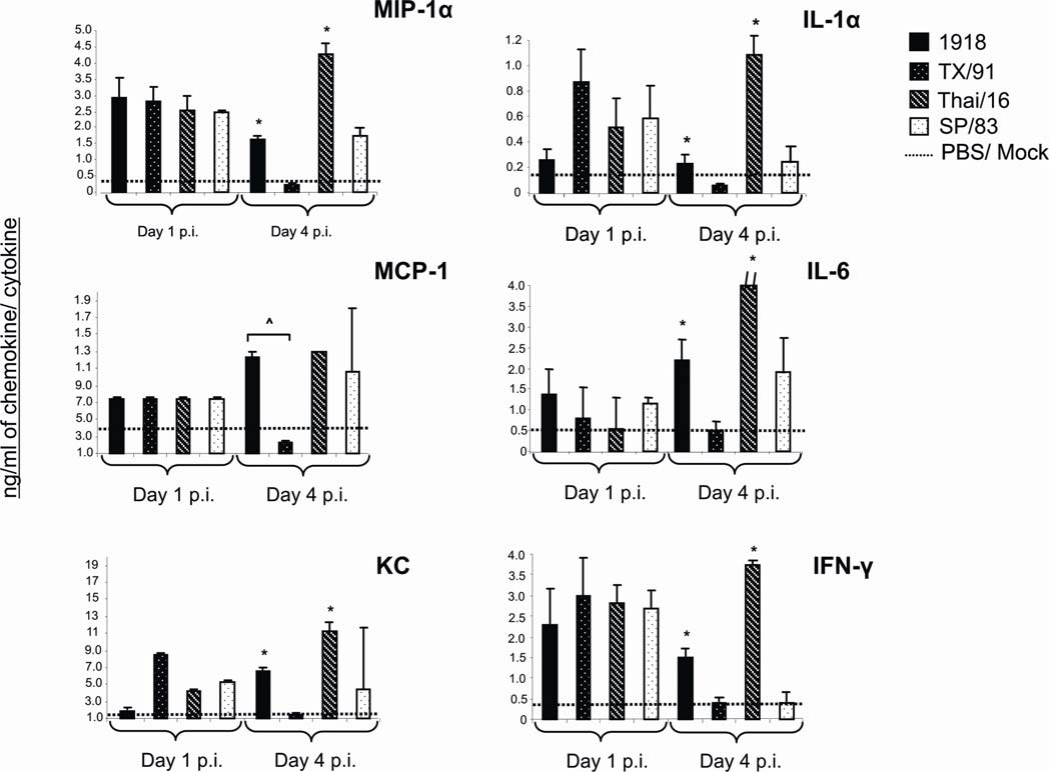
Lung cytokine response. Cytokine levels from infected lungs (n = 3 mice per virus group, days 1 and 4 post-inoculation (p.i.)) were measured individually and in duplicate by the Bioplex Protein Array system lungs. Baseline cytokine levels from PBS inoculated mice (mock) are shown as a dashed line in each cytokine graph. Bars represent means of 3 mice from each infection group±standard deviation (SD). Protein levels of IL-6 in Thai/16 infected animals exceeded the y scale shown (indicated by a dash//, the concentration in Thai/16 infected mice was 11.7 ng/ml). ˆ *p*<0.05 between 1918 and TX/91 viruses, * *p*<0.05 between 1918 and Thai/16 (HP) virus groups and SP/83 and TX/91 (LP).

To better understand potential influences on immune cell population dynamics in the lung following HP influenza virus infection as demonstrated in Figure 2B and Table 2, we analyzed 17 chemokines and cytokines in the lungs of mice on days 1 and 4 p.i and report data here on 6 of the analytes that revealed significant differences among the viruses tested (Figure 3). These earlier time points were chosen in an effort to understand the temporal relationship with lung immune cell infiltration in 1918 and Thai/16 infections (Figure 2) and day 4 p.i. is a time when significant differences were observed in lung virus titers between HP and LP infection groups (Figure 1). As shown in Figure 3, on day 1 p.i. TX/91 infected lungs exhibited higher titers of IL-1α, IFN-γ, and KC compared to 1918 virus-infected lungs though levels of MIP-1α, MCP-1, and IL-6 were higher in 1918 virus infected lungs. Cytokine levels were similar between the H5N1 viruses at day 1 p.i.. In contrast, lung tissue cytokine and chemokine levels at day 4 p.i. were higher among 1918 and Thai/16 virus-infected mice compared to those infected with the subtype-matched LP (TX/91 and SP/83) counterpart viruses for all cytokines and chemokines measured. Protein levels of the potent monocyte chemoattractant MCP-1 were 10-fold higher in 1918 and H5N1 virus-infected lungs than TX/91 virus-infected lungs, whereas levels of the MIP-1α chemokine were notably elevated in Thai/16 virus-infected lungs. KC (the mouse equivalent of human IL-8) [20],[23] levels were 10-fold higher in Thai/16 and 3-fold higher in 1918 virus infected lungs compared to TX/91 virus-infected lungs with SP/83 levels similar to 1918 infected lungs. The HP viruses were also potent inducers of IL-1α and IFNγ. IL-6, (a generally pro-inflammatory cytokine) was also elevated in 1918 and H5N1 virus-infected lungs compared to TX/91 levels. As shown in Figure 3, significant differences (ˆ *p*<0.05) were measured between 1918 and TX/91 virus infected lungs on day 4 p.i. for each cytokine and chemokine, accentuating important immunological differences in the immune response to the H1N1 pandemic virus versus a contemporary H1N1 virus.

### Innate immune cells are targets of highly pathogenic influenza virus infection

**Figure 4:**
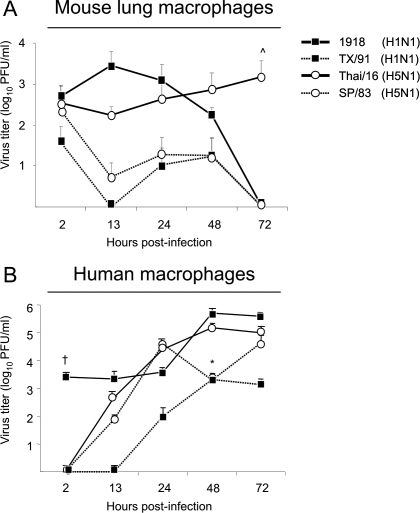
Growth of viruses in primary macrophages. Primary macrophages were harvested from the lungs of healthy BALB/c mice through tissue digestion (A) and developed from human peripheral blood monocytes (B) and infected *in vitro* (MOI = 0.1) with influenza viruses as described (Table 1, Methods). Mouse lung macrophages were grown in 12 well plates and infected with viruses in duplicate and the supernatants sampled for virus growth. Human macrophages were grown in 6 well plates and also infected in duplicate. Virus titers were determined from supernatants in duplicate by plaque assay on MDCK cells. Graphs are representative of results obtained from three independent infection experiments. ˆ *p*<0.05 between Thai/16 and 1918, SP/83 and TX/91 viruses 72 hrs p.i (A). * *p*<0.05 between HP Thai/16 and 1918, and LP SP/83 and TX/91 viruses 48 hrs p.i. (B). ^†^ *p*<0.05 between the 1918 virus and other viruses 2 hours post- infection (B).

Due to the increased presence of macrophages in the lungs following HP influenza virus infection, replication of paired H1N1 and H5N1 viruses (Table 1) was assayed over time in primary human PBMC-derived macrophages and mouse lung macrophages to address whether these cells are specific targets of viral infection and can productively replicate HP viruses. Mouse lung macrophages were harvested from naive BALB/c mice and infected *in vitro* (MOI = 0.1, Figure 4A) as described. While the HP 1918 and Thai/16 viruses exhibited a slight increase in log titer very early after inoculation (13–24 hrs p.i.), overall these viruses did not replicate efficiently compared to the growth kinetics of these viruses observed in lung epithelial cells [22],[24]. At 13 hrs p.i. there was 18 fold higher 1918 and Thai/16 virus released from infected cells than TX/91 and SP/83 virus-inoculated cultures and nearly 54 times more 1918 and Thai/16 virus than TX/91 or SP/83 virus at 24 hrs p.i. While virus titers in the 1918, TX/91 and SP/83 infection groups declined after the 48 hr time point, the Thai/16 infected macrophages maintained virus titers and these differences were statistically significant (ˆ *p*<0.05) at 72 hrs p.i.. The lack of prolific replication of these four viruses in primary mouse lung macrophages was confirmed further when a higher MOI (1.0) was utilized (data not shown). Human macrophages also supported replication of all four viruses (Figure 4B, MOI = 0.1). At 48 hrs p.i. 1918 and Thai/16 infected macrophages exhibited 180 times higher virus titers than TX/91 and SP/83 infected cultures. Interestingly, 1918 virus-infected macrophages exhibited a higher baseline titer soon following infection that was found to be statistically significant when compared to the other infection groups (^†^ *p*<0.05). In summary, while human macrophages are a target of viral replication and support replication well, mouse lung macrophages support low levels of 1918 and Thai/16 virus production early following infection.

**Figure 5:**
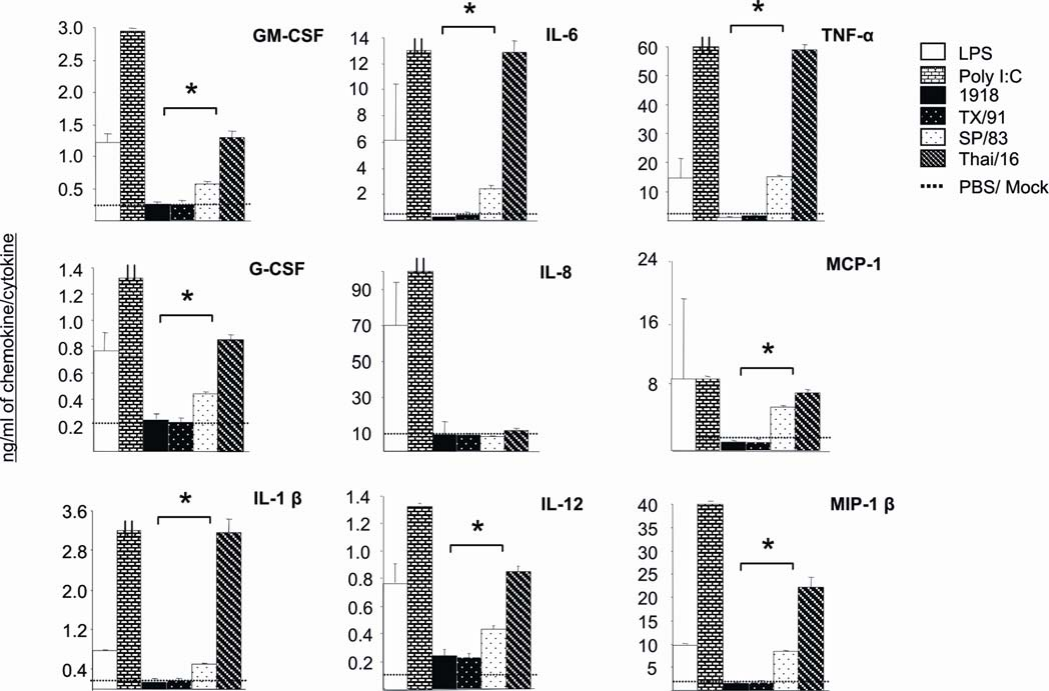
Cytokine response from infected human macrophages. Primary macrophages were developed from human peripheral blood monocytes in 6 well plates and infected *in vitro* (MOI = 0.1) with influenza viruses in duplicate as described (Methods, Table 1). Supernatants were sampled 48 hours post-infection and cytokines measured by Bioplex Protein Array assay (Methods). Macrophages were also treated with bacterial lipopolysaccharide (LPS, 100 ng) and Poly I/C (100 ng) to serve as positive inducers. * *p*<0.05 between H5N1 and H1N1 viruses.

Additional experimentation with primary human macrophages revealed that levels of pro-inflammatory cytokines were higher for H5N1-infected cells than either 1918 or TX/91 virus infected cells (48 hrs p.i., MOI = 0.1, Figure 5). Significant differences in cytokine levels were observed between H5N1 and H1N1 virus infected macrophages in all cytokines measured (*p<0.05) except IL-8 where all four viruses elicited similar levels of this chemokine. Interestingly, the 1918 virus elicited similar cytokine responses as to TX/91 inoculated cells in every cytokine measured even though 1918 virus titers at this time point post-infection were 2.5 logs greater than TX/91 infected macrophages (Figure 4B). Thai/16 infected cultures elicited at least a 2 fold greater cytokine response than the SP/83 virus infected cultures in every cytokine measured except IL-8 and MCP-1 chemokines where Thai/16 levels were only slightly higher than SP/83 levels.

**Figure 6:**
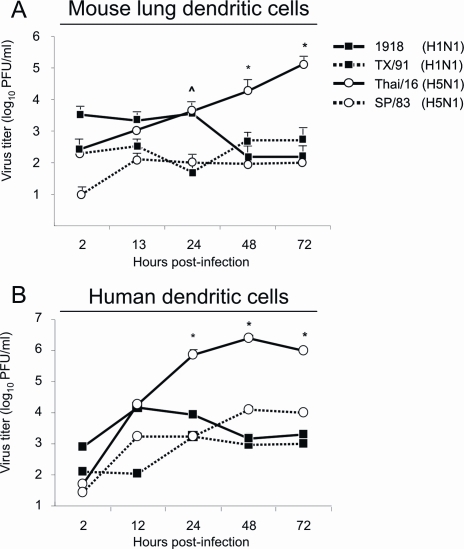
Growth of viruses in primary dendritic cells. Primary dendritic cells were isolated from the lungs of healthy BALB/c mice and developed from human monocytes as described in Methods and infected *in vitro* with LP and HP influenza viruses (Table 1). Growth of H5N1 and H1N1 viruses in mouse lung macrophages (A, MOI = 0.1) and in human monocyte-derived dendritic cells (B, MOI = 1.0). (ˆ *p*<0.05 between HP Thai/16 and 1918 viruses and LP SP/83 and TX/91 viruses. * *p*<0.05 between Thai/16 and 1918, SP/83 and TX/91 viruses.) Graphs are representative of results obtained from three independent experiments.

Because of their important role in antigen presentation to T cells, we also assessed the ability of primary dendritic cells to productively replicate pandemic H1N1 and human H5N1 viruses (Figure 6A and B). While the HP Thai/16 H5N1 virus replicated slowly over time in cultured mouse lung dendritic cells (Figure 6A), these cells failed to productively replicate 1918, TX/91 and SP/83 viruses. Significant differences in virus titers were measured between HP (1918 and Tha/16) infected cells and TX/91 and SP/83 infected cells at 24 hrs p.i. (Figure 6A, ˆ *p*<0.05). Significant differences in virus titers was also observed between Thai/16 inoculated cultures and the other infection groups at 48 and 72 hrs p.i. (* *p*<0.05). Similar to infected human macrophages, these trends were conserved when primary human dendritic cells were the target of infection with these viruses (Figure 6B). Significant differences in virus titers was observed between Thai/16 inoculated cultures and the 1918, TX/91, and SP/83 infection groups at 24, 48 and 72 hrs p.i. (* *p*<0.05).

**Figure 7:**
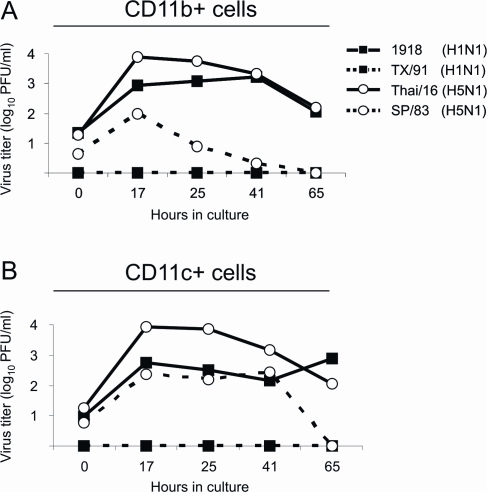
Virus replication from primary lung macrophages and dendritic cells cultured *ex vivo*. BALB/c mice were infected intranasally with 10^2^ PFU of the indicated viruses. Three days post-inoculation, lungs were removed from 2–3 mice per virus group. Cell suspensions were prepared from pooled samples and macrophages (A) and dendritic cells (B) were isolated by CD11b+ ad CD11c+ MACS column purification, respectively. Supernatants from cultures were sampled over time to measure virus production. Virus was titered in duplicate by plaque assay.

To determine if innate immune cells are being productively infected *in vivo*, macrophages and dendritic cells were purified from lungs of infected mice and cultured for infectious virus. Lungs from infected (3 days p.i.) mice were harvested and e*x vivo* cultures containing either lung macrophages or dendritic cells were sampled for infectious virus over a 65 hour time period. While the seasonal influenza isolate, TX/91 virus was not produced from either macrophages (CD11b+) or dendritic (CD11c+) cells, the 1918 pandemic virus as well as the two human H5N1 isolates were released into the culture supernatant (Figure 7), indicating that these cells are being productively infected in the mouse lung. In CD11b+ (macrophages) cell cultures (Figure 7A), the 1918 and Thai/16 virus infected cells released more infectious virus over time than the LP SP/83 virus infected culture. In CD11c+ (dendritic) cell cultures, the Thai/16 virus infected cultures released more infectious virus than the 1918 and SP/83 virus infected cultures (Figure 7B). These data further demonstrate that mouse lung macrophages and dendritic cells are susceptible to highly pathogenic influenza virus infection in the lung tissue.

## Discussion

Lung consolidation has been described as a pathological feature of severe influenza virus infection caused by the 1918 pandemic virus and H5N1 viruses in humans [9],[25],[26] as well as in animal models [11],[12],[18]. Using a detailed flow cytometry evaluation, the current study set out to characterize differences in the cellular innate immune response in the mouse lung following highly pathogenic (HP) or low pathogenicity (LP) influenza virus infections. Lungs from mice infected with the HP 1918 H1N1 virus and a recent H5N1 human isolate (A/Thailand/16/04 (Thai/16)) exhibited a significant increase in cellularity in comparison to the LP seasonal H1N1 isolate, A/Texas/36/91 (TX/91). Significant differences in titer between HP and LP virus-infected mice were observed as early as day 1 post-inoculation (p.i.), likely forecasting the dramatic increase in immune cell infiltration in the lungs of these virus-infected mice. Interestingly at day 7 p.i. when peak lung cellularity was observed in HP virus infection groups, differences in virus titers between paired subtype viruses (1918 compared to TX/91 and Thai/16 compared to SP/83) were minimal and were limited to a maximum difference of 1 log (Figure 1), indicating a failure by the immune system to clear the viral infection. Lung cellularity was further investigated by characterizing the comprising immune cell sub-populations. We observed a significant increase in macrophages and neutrophils early following infection with the 1918 and Thai/16 viruses, and their sustained presence in the lung tissue mark a distinction between HP and LP influenza virus infection. These data show that virus replication in the lungs of HP influenza infections are sustained at high levels the first week of infection regardless of the high numbers of immune cells present in the tissue. Although the current study did not determine whether these cells are playing an antiviral role against the HP viruses, it has been previously shown that neutrophils and macrophages assist in the clearance of influenza virus early during infection; these cells appear to be capable of only partially reducing the virus load in the lung despite their presence at high numbers [20]. We also observed a decrease in the percentage of lung–associated dendritic cells and T cell lymphocytes during HP influenza virus infection. A decrease in the number of circulating lymphocyte populations has also been previously observed in the peripheral blood of humans and mice infected with H5N1 viruses [5],[9],[10],[15],[20],[27]. The precise mechanism of leukocyte depletion during H5N1 infections is not well understood, but evidence of apoptosis in the spleen and lungs of HP H5N1-infected mice detected *in situ* suggests a mechanism for cell loss [16].

Influenza virus growth in the respiratory epithelium and the subsequent release of chemotactic proteins from those cells may encourage the increased presence of macrophages and neutrophils [28],[29]. Macrophages and neutrophils can secrete chemokines and cytokines that can act in an autocrine fashion which in turn can promote the increased migration of those cells and other leukocytes into the lung tissue [30]. Elevated levels of certain chemokines and cytokines have been associated with high viral load and severe disease in H5N1 virus infected patients [26],[31]. These studies show that infection with HP 1918 and Thai/16 H5N1 viruses result in elevated amounts of pro-inflammatory chemokines and cytokines in the lungs of mice day 4 post-infection compared with TX/91 and SP/83 infected mice, a time point that correlates with rising but significantly different lung virus titers between infection groups. Elevated levels of the chemokines MCP-1 [32] and MIP1-α were observed among H5N1 and 1918 virus infected mouse lungs. Although, MIP-1α does not appear to be critical for virus replication and spread in the mouse model [10], this chemokine exhibits a variety of pro-inflammatory activities including macrophage and neutrophil recruitment and has been associated with fatal outcomes in human H5N1 virus infections [31]. Lungs infected with the 1918 pandemic and Thai/16 H5N1 viruses also exhibited significantly higher levels of IFN-γ on day 4 p.i compared to their subtype-paired LP virus counterparts. IFN-γ is known to mediate the increased production of nitric oxide [33] which can subsequently result in the recruitment of more neutrophils and macrophages. Higher levels of IL-6 were measured in 1918 and Thai/16 virus infected lungs, supporting observations obtained with the 1997 H5N1 viruses [10] and has been correlated with systemic illness symptoms and fever in experimental human TX/91 infections [34]. By directly measuring cytokine protein levels, these data provide confirming evidence of a heightened lung cytokine response to 1918 and H5N1 infection in mice [13].

Interestingly, while we reveal marked similarities between the HP 1918 and Thai/16 viruses in overall lung cellularity, virus growth and patterns of immune cell sub-population dynamics over time, Thai/16 virus infection consistently resulted in higher levels of chemokines and cytokines both in mouse lungs and human macrophages. Although it has been shown recently by two independent research groups that the lack of key cytokines (through the use of single cytokine gene knockout mice) had no effect on the overall disease outcome or virus replication among H5N1 virus-inoculated mice [10],[35], the present results along with data from others continue to indicate that pro-inflammatory cytokines correlate with disease outcome [36]. It is thought that these immune mediators do not act singularly *in vivo* and it will be critical to reveal these concerted interactions (both locally in the lung and systemically) to further our understanding of the pathogenesis of H5N1 infection in animal models and in human patients.

Macrophages and dendritic cells play a fundamental role in the lung at all stages of influenza virus infection [20],[37],[38]. We have provided evidence regarding the higher replication efficiency of HP influenza viruses in primary human macrophages and dendritic cells, a property that has also been demonstrated previously in other primary cells [24],[39]. Mouse macrophages were also susceptible to virus infection *in vitro*, however they did not support productive replication to the level observed in primary human monocyte-derived macrophages (Figure 4B) or lung epithelial cells [21],[24]. However, the cytopathic effects (estimated by visual examination of monolayers) among HP virus-infected mouse and human macrophages was observed in a shorter period of time compared to LP virus infected cells. Interestingly, the 1918 virus exhibited higher baseline titers in human and mouse macrophages as early as 2 hrs p.i. compared to the TX/91 H1N1 virus or the H5N1 viruses, indicating a curious property of this pandemic virus. While the importance of the binding properties of the HA molecule has been demonstrated elsewhere extensively [22],[40],[41], further resolution of this interesting finding and its immunological importance deserves further experimentation. The role of other cell surface molecules on innate immune cells such as the mannose receptor in HP influenza infection should be investigated [42],[43]. The higher viral replication of HP viruses in dendritic cells also correlated with the severe pulmonary disease observed in mice. We also demonstrated definitively that these cells are targets of infection *ex vivo* by highly pathogenic influenza viruses like the 1918 pandemic virus and recent H5N1 isolates (Figure 7). Thus, it appears that macrophages and dendritic cells may contribute to the pathogenesis of HP virus infection due to their susceptibility to influenza virus infection. An inability to mount an adaptive immune response due to direct infection of important innate immune cells such as macrophages and dendritic cells may be a critical difference in host outcome during influenza virus infection [44]. This coupled with the phenomenon of T cell depletion in infected mice [17] and human patients [9],[31] may allow for uncontrolled viral replication. While reducing viral load through anti-viral intervention remains the best treatment option for H5N1 patients, therapies that moderate immunopathology may help to reduce the high case fatality rate currently associated with virus infection [45].

## Materials and Methods

### Viruses and cells

All *in vitro* and *in vivo* experiments were performed under the guidance of the U.S. National Select Agent Program in negative pressure HEPA-filtered biosafety level laboratory (BSL-3+) enhanced laboratories and with the use of a battery powered Racal HEPA-filter respirator and according to Biomedical Microbiological and Biomedical Laboratory procedures. Influenza viruses used in these experiments included the reconstructed A/South Carolina/1/18 virus (H1N1) [21], A/Texas/36/91 (H1N1), A/Thailand/16/2004 (H5N1), and A/Thailand/SP/83/2004 (H5N1). These viruses were chosen as pairs within subtypes due to their respective fifty percent lethal dose (LD_50_) titers in mice and ferrets (Table 1). Low pathogenicity in this manuscript refers to the non-lethal phenotype of the seasonal H1N1 TX/91 virus and the low virulence SP/83 H5N1 isolate [18],[21]. The Thai/16 and SP/83 viruses differ from each other in 13 amino acids in 7 proteins and this sequence comparison has been published previously [18]. The A/Texas/36/91 and H5N1 viruses were grown in 10 day old embryonated hen's eggs and the 1918 viruses grown in MDCK cells. All virus stocks were titered by plaque assay on MDCK cells prior to mouse infections.

Human peripheral blood monocytes (PBMC's) were obtained by Histopaque (Sigma-Aldrich, St. Louis, MO) density gradient centrifugation of whole blood donated by healthy donors aged 20–40 yrs old without history of influenza vaccination in the past year. Whole blood was obtained through an approved protocol by both the Emory University Institutional Review Board (IRB) and CDC IRB (Emory University Hospital Blood Bank is an FDA-accredited Blood Bank). Human monocytes were obtained by negative selection column enrichment (Miltenyi Biotech, Auburn, CA) yielding approximately 90% CD14+ purity as determined by FACS analysis. For development of macrophages, monocytes were cultured at 37°C in 6 well plates in Macrophage SFM media (Gibco, Grand Island, NY) with 20% heat inactivated autologous serum for 7 days in the presence of GM-CSF (1000 U) before infection [46]. Human macrophages cultured in this manner typically displayed classical morphology with the phenotype: CD11b^low^, CD11c^low^, HLA-DR^low^, CD14^low^, CD40^low^, CD80^low^, CD83^low^, CD86- (Figure 4C and D). For the development of dendritic cells (DC's), monocytes were grown in RPMI (Gibco) with 20% heat inactivated autologous serum for 10 days in the presence of IL-4 (1000U) as previously described [46]. Dendritic cells developed in this manner typically displayed a classical morphology with the presence of dendritic processes with the phenotype: CD11b^low^, CD11c^high^, HLA-DR^high^, CD14^low^, CD40^high^, CD80^high^, CD83^high^, CD86^high^ (Figure 6B).

To obtain primary mouse lung macrophages and dendritic cells, lungs from naïve mice were removed and tissue disrupted as described above through the use of collagenase digestion and cell suspensions prepared. Macrophages (CD11b+) and dendritic cells (CD11c+) were extracted from contaminating cells by selection on magnetic columns (Miltenyi Biotech, Auburn, CA). Cells were washed twice with media containing 20% FCS (Macrophage SFM for macrophages (Gibco) or RPMI (Gibco) for dendritic cells) and cultured for 24 hours before in vitro infection. Primary mouse lung macrophages typically displayed the phenotype: CD11b^high^, CD11c-, MHCII^high^, CD40^high^, CD80^high^, CD83^high^ (Figure 4A and B). Primary mouse lung dendritic cells typically displayed typical morphology with dendritic extensions and the phenotype: CD11b-, CD11c^high^, MHCII^high^, CD40^high^, CD80^high^, CD83^high^ (Figure 6A).

### *In vitro* infections

Primary human and mouse cells were washed 3× with serum free growth media and infected for 1 hour with viruses (Table 1) at a multiplicity of infection (MOI) of 0.1. Following infection, cells were washed 3× with serum free growth media and 1 ml SFM media, containing 1 µg/ml of TPCK-treated trypsin (Sigma-Aldrich), was placed into the wells. Virus growth was measured over time in triplicate wells for each experiment and titered in duplicate by standard plaque assay on MDCK cells. All macrophage and dendritic cell data reflects at least three independent experiments (Figures 4 and 6). Cytokine levels produced from infected human macrophages (MOI = 0.1) were quantitated 48 hrs p.i. by BioPlex assay (Figure 5). Escherichia coli lipopolysaccharide (LPS, 100 ng, Sigma-Aldrich) and Poly I/C (100 ng, Sigma-Aldrich) were used as positive control stimulants.

### Mice infections

All animal research was conducted under the guidance of CDC's Institutional Animal Care and Use Committee and in an Association for Assessment and Accreditation of Laboratory Animal Care International- accredited facility. 8–10 week old female BALB/c mice (Harlan, Indianapolis, IN) were anesthetized with Avertin [20] (Sigma-Aldrich) and infected intranasally (i.n.) with 50 µl of 10^2^ PFU of influenza viruses prepared in phosphate buffered saline (PBS). Avertin was chosen as the anesthetic because it provides consistent mouse infections with the viruses used in these studies. Using the sublethal (10^2^ PFU) inoculum, 1918 and Thai/16 virus infected mice survive a prolonged disease course allowing for the measurement of the influx of inflammatory cells into the lung tissue during a full course (∼7–9 days) of influenza virus infection. At indicated times post-infection (n = 3 mice per virus group) mice were euthanatized and exsanguinated. Lungs were removed from individual mice without PBS perfusion and included total lung cell counts included cells located in the bronchoalveolar airways. Perfusion was not possible in many cases of HP influenza virus infection due to the presence of microvascular hemorrhage. We obtained similar results when performing these lung cell quantitation assays with or without lung perfusion in LP virus infected mice and therefore did not introduce this variable in our high containment laboratory. Whole lung cell suspensions were prepared in Dulbecco's minimal essential media (DMEM) with 20% fetal calf serum following collagenase-DNase treatment and manual disruption [47]. Red blood cells were removed by lysis buffer treatment (Sigma-Aldrich). Total viable lung cell number was determined for each mouse by trypan blue exclusion on a hemocytometer.

For the *ex vivo* experiment, macrophages and dendritic cells were isolated from the lungs of infected BALB/c mice. Two or three mice were infected i.n. with 10^2^ PFU of each of the four viruses described in this study (Table 1). Three days post-inoculation, lungs were removed without perfusion and cell suspensions were prepared as described above. Lungs were pooled from mice in each virus infection group. Macrophages and dendritic cells were isolated by positive selection on CD11b+ or CD11c+ MACS columns. Columns containing bound immune cells were washed extensively (5x) and CD11b+ or CD11c+ cells were eluted off the magnetic columns and cultured in 6-well plates in 5 ml of RPMI containing 5% BSA. Supernatants were collected at the indicated times and virus content was determined in a standard plaque assay on MDCK cells.

### Flow cytometry and lung immune cell quantitation

Lung cell suspensions were incubated with anti-Fc block (anti-mouse CD16/CD32) to reduce non-specific antibody binding for 10 min. prior to staining for 1 hr with fluorophore-conjugated antibodies (BD Biosciences, San Diego, CA) specific for immune cell populations according to standard protocols [48] and included: CD11b-PE (pan-macrophage), CD11c-APC (pan-dendritic cell), Ly6G/C-FITC (neutrophil), CD4-PE and CD8-APC T cell markers (Table 2, Figure 2). Cells were washed twice with PBS and fixed overnight at 4°C with 2% paraformaldehyde. Samples were safety tested for infectious virus and removed from the BSL3+ laboratory. Flow cytometry was performed on a FACSAria flow cytometer (BD Biosciences). To further characterize primary mouse and human macrophage and dendritic cells we utilized the following fluorescently conjugated antibodies for flow cytometric analysis: MHC II (I-A/I-E)-PE, HLA-DR-PE, CD14-FITC, CD40-FITC, CD80-FITC, CD83-APC, and CD86-APC (BD Biosciences) (Figures 4 and 6).

### Virus titrations and cytokine analysis

At various times post-infection (n = 3 mice per virus group) lungs were removed and stored at −70°C until virus and cytokine levels could be quantified. Lungs were homogenized individually in 1 ml PBS. Virus was titered from clarified lung homogenates by standard plaque assay on MDCK cells in duplicate and titers are reported as plaque forming units per ml PBS (PFU/ml, Figure 1). Cytokine protein levels were measured (day 4 post-infection ( p.i.)) by the Bioplex Protein Array system [49] (Bio-Rad, Hercules, CA) using beads specific for mouse G-CSF, IL-1α , IL-1β, IL-3, IL-6, IL-9, IL-12 (p40), IL-12 (p70), IL-13, Eotaxin, TNFα, RANTES, KC, MIP1-α, MIP-1β, MCP-1, and IFN-γ. Cytokine protein levels were measured according to the manufacturers instructions by fluorescently conjugated monoclonal antibodies in duplicate against a standard curve (Figures 3 and 5).

### Statistical analysis

Statistical significance of differences between experimental groups was determined through the use of the unpaired, non-parametric Student's *t* test. Values of *p*<0.05 were considered significant.
